# Baseline Depressive Symptoms, Completion of Study Assessments, and Behavior Change in a Long-Term Dietary Intervention Among Breast Cancer Survivors

**DOI:** 10.1007/s12160-015-9716-1

**Published:** 2015-06-20

**Authors:** Julie B. Wang, John P. Pierce, Guadalupe X. Ayala, Lisa A. Cadmus-Bertram, Shirley W. Flatt, Hala Madanat, Vicky A. Newman, Jeanne F. Nichols, Loki Natarajan

**Affiliations:** University of California, San Diego, 530 Parnassus Avenue, Library Room 366, San Francisco, CA, 94143 USA; University of California, San Diego, Family Medicine and Public Health, 9500 Gilman Drive, MC 0901, La Jolla, 92093-0901 CA USA; University of California, San Diego, Moores Cancer Center 3855 Health Sciences Drive, MC 0901, La Jolla, CA, 92093-0901 USA; San Diego State University, Graduate School of Public Health, 5500 Campanile, MC 4124, San Diego, CA 92182-4124 USA; San Diego State University, Institute for Behavioral And Community Health, 9245 Sky Park Court, Ste. 221, San Diego, CA 92123-4311 USA; University of Wisconsin – Madison, Department of Kinesiology, 2000 Observatory Drive, Madison, WI 53706 USA; San Diego State University, Graduate School of Public Health, 5500 Campanile MC 4162, San Diego, CA 92182-4162 USA; San Diego State University, Institute for Behavioral And Community Health, 9500 Gilman Drive, MC 0811, La Jolla, CA 92093-0811 USA

**Keywords:** Dietary change, Depressive symptoms, Adherence, Behavioral activation

## Abstract

**Background:**

Depressive symptoms can lower adherence and change in dietary studies. Behavioral activation may reduce these effects.

**Purpose:**

This study aims to assess relationships among depressive symptoms on adherence and dietary change in the Women’s Healthy Eating and Living (WHEL) Study

**Methods:**

Secondary analyses from the WHEL Study, which achieved major dietary change in breast cancer survivors (*N* = 2817), were conducted. Logistic regressions were undertaken of baseline depressive symptoms (six-item Center for Epidemiologic Studies Depression Scale (CES-D)) with (1) completion of 1- and 4-year study assessments and (2) validated change in dietary behavior in the intervention group.

**Results:**

In the comparison group (vs. intervention), depressive symptoms lowered completion of dietary recalls and clinic visits [4 years: odds ratio (OR) = 2.0; 95 % confidence interval (CI) = 1.4–3.0]. The behaviorally oriented intervention achieved major change in those furthest from study targets, although changes were lower in those with depressive symptoms: fruit/vegetable (+37.2 %), fiber (+49.0 %), and fat (−22.4 %).

**Conclusions:**

Behavioral activation in dietary change interventions can overcome the impact of depressive symptoms.

## Introduction

Increasing consumption of plant-based foods and reducing energy from fat are public health goals to reduce the incidence of a number of diseases including both cardiovascular disease and cancer [1]. However, affective disorders such as depression and anxiety have considerable potential to confound the results in dietary change studies [2]. Approximately one fifth of female breast cancer survivors who join behavioral research studies have depressive symptoms [3, 4], and these women are more likely to have lower dietary quality at baseline [5]. They are less likely to complete study assessments, and those who do complete are less likely to be responsive to the intervention [6, 7]. Specific strategies are needed in studies to minimize this differential response in such a large subpopulation.

A popular therapeutic framework for treating depression is *behavioral activation*. In this framework, depressive symptoms develop following periods of negative mood and behavioral avoidance, which are associated with a low rate of response-contingent positive reinforcement [8]. This low rate of positive reinforcement could occur due to a combination of the following four conditions: (1) decreased number of reinforcing events, (2) decreased availability of reinforcers, (3) deficits in behavior needed to experience reward, and (4) increased experience of aversive/punishing stimuli. Interventions containing behavioral activation strategies focus on overcoming such conditions [9].

The Women’s Healthy Eating and Living (WHEL) Study investigated the role of dietary patterns in outcomes following diagnosis of breast cancer. The key behavior change intervention in this long-term randomized trial was a client-centered telephone counseling protocol that was guided by social cognitive [10] and prospect theories [11]. The protocol [12] emphasized the primacy of self-efficacy throughout the behavior change process and the importance of framing performance to enhance efficacious judgments. The intervention used positive reinforcement as a key strategy. Personal counselors assisted participants to implement self-regulatory skills, set proximal goals, and monitor and judge performance. Serial proximal dietary goals were encouraged to be challenging yet achievable, providing participants many opportunities to celebrate small successes throughout the project. The study used an innovative extrinsic reward system to motivate all participants to complete study assessments. The WHEL intervention was successful in achieving and maintaining long-term change in dietary intake across the overall study population [13]; however, the study has not reported on differential effects in women with depressive symptoms.

In this paper, we conducted secondary analysis of data from the WHEL Study to examine the relationship between depressive symptoms at baseline with adherence to study assessments and dietary change. We hypothesized that (1) women with depressive symptoms would be further from the study dietary targets at baseline than those without such symptoms; (2) women with depressive symptoms would complete fewer follow-up dietary assessments and clinic measurement visits than those without such symptoms, and these differences would be greater in the comparison group compared to the intervention group; and (3) in the intervention group, women with depressive symptoms would achieve improvements in dietary change, but these changes would be smaller than those in women without depressive symptoms.

## Methods

### Study Population

The WHEL Study recruited 3088 cancer-free women within 4 years of an early-stage breast cancer diagnosis from seven centers in the south and west regions of the USA between 1995 and 2000 [12, 13]. Invitational letters were sent to potentially eligible women identified from cancer registries or medical record review. This screening pool was increased by 30 % through community advertising. Of the 7532 screened, 41 % met full eligibility criteria and were randomized. Overall, approximately 20 % had depressive symptoms at baseline, which was comparable to similar non-breast cancer populations [6, 14] and such symptoms were unrelated to breast cancer characteristics [15]. For this analysis, we excluded 191 women who were not eligible for the 4-year study assessment (e.g., had an early study event) resulting in a sample size of 2817.

### WHEL Study Intervention

The WHEL Study intervention targeted dietary pattern had been pre-tested for feasibility in this population [16] and consisted of daily intakes of 5 vegetable servings, 16 oz of vegetable juice, 3 fruits, 30 g of fiber, and 20 % energy from fat [12]. The study did not promote weight loss or increased physical activity in order to assess the independent effect of dietary change. In the first year, the WHEL intervention included telephone counseling, 12 cooking classes, and regular newsletters and print materials aimed at maintaining motivation. The intervention included several activities early in the intervention to capture the participants’ initial enthusiasm and to build motivation for behavior change: these included a 2-h orientation session with a slide presentation and food demonstration, outlining the study goals and rationale for the cancer-preventive dietary pattern; the first telephone call with the assigned counselor also focused on building motivation and sought a personal commitment to making the targeted changes [17].

The counseling protocol followed three phases of decreasing intensity aimed at helping the participant use her self-regulatory skills to explore how much she could change her dietary pattern and then to consolidate as much change as possible into her daily lifestyle. The initial counseling phase was tailored to the individual (three to eight 45-min calls in the first 4 to 6 weeks) and began with a 24-h dietary recall to provide the basis to discuss recent dietary performance and to set proximal goals. Throughout each call, the counselor took every opportunity to provide positive reinforcement and encouragement. The second phase aimed for weekly calls for 9 weeks in which counselors and participants reviewed use of the study’s validated self-monitoring tool for assessing performance on targeted dietary intake, addressed barriers, and developed strategies to integrate dietary change into participants’ lifestyles. Again, counselors focused on helping participants to positively view their dietary performance. A relapse prevention phase, focused on maintaining motivation and preventing setbacks, began at about month 4, with 9 monthly calls, followed by quarterly calls through study completion. Participants in the WHEL intervention received an average of 31 calls over these 6 years.

### Comparison Group

Women randomized to the comparison group were provided printed dietary guidelines from the United States Department of Agriculture (USDA) and the National Cancer Institute (NCI) and were invited to four cooking classes on topics unrelated to the study’s dietary pattern (e.g., calcium intake) and received monthly study newsletters (with topics unrelated to the intervention’s dietary pattern). Specifically, the USDA and NCI dietary guidelines were 5 daily servings of fruits and vegetables, 20 g of fiber, and 30 % energy from fat [16, 18].

### Study Assessments and Clinic Visits

The WHEL Study included five separate sets of assessments around the anniversary of study enrollment (thus ensuring equal distribution of measures by months of the year), each including multiple 24-h dietary recalls (telephone based), clinic measurement visits, questionnaires, as well as half-yearly telephone check-ins [12]. In this study, we focused on the two most critical for assessing dietary change: the set of 24 h dietary recalls and the clinic measurement visit at the year 1 and year 4 time points. The study used a *frequent flyer*-type point program to incentivize participants to complete scheduled study assessments. Points were assigned according to the importance of the assessment and awarded to participants’ *accounts*. Both intervention and comparison groups had the ability to accrue the same number of points for completing assessments. Participants redeemed their points through gift certificates that were donated from Walmart and Macy’s, or they could use their points to enter a yearly raffle for a chance to win two round-trip tickets from Southwest Airlines for travel in the USA. The protocol was approved by the institutional review boards at each center.

#### Twenty-Four-Hour Dietary Recalls

Self-reported dietary pattern was assessed over the telephone at regular intervals throughout the study [12, 19]. Trained assessors, blinded to study group, scheduled dietary assessment calls from 4 randomly selected days, stratified by week and weekend days, within a 3-week window around the anniversary of study enrollment. All dietary assessments used the multiple-pass and multiple-probe approach consistent with the Minnesota Nutritional Data System software (NDS-R, 1994–2006). In this system, key dietary components were estimated using standardized food tables.

#### Clinic Visits

Participants were invited to schedule a clinic measurement visit at a time close to their dietary recalls. These visits included measurement of anthropometrics, blood pressure, and the collection of a venipuncture blood sample, which was separated and stored at −80 °C. The study has reported that neither study group had significant weight loss over the study period [13].

#### Depressive Symptoms

We used the six-item short form of the Center for Epidemiologic Studies Depression Scale (CES-D) to assess depressive symptoms [20–22]. Participants rated how often in the past week (1) you felt depressed, (2) your sleep was restless, (3) you enjoyed life, (4) you had crying spells, (5) you felt sad, and (6) you felt that people disliked you. Items were scored as 0 = rarely or none of the time (<1 day), 1 = some or a little of the time (1–2 days), 2 = occasionally or a moderate amount of the time (3–4 days), and 3 = most or all of the time (5–7 days). Item 3 was reversely scored. All items were summed to calculate a depressive symptom score. The standard threshold value ≥5 was used to define level of depressive symptoms [20–24].

#### Measurement of Blood Carotenoid Concentration

Plasma carotenoid concentrations are known biomarkers of fruit and vegetable intake [18]. In previous analyses, using plasma carotenoids as the gold standard, we identified that the validity of self-reported carotenoid intake through vegetables and fruit was not high and was different across study groups [19, 25]. Accordingly, in this paper, we use the carotenoid measure as our marker of change in vegetable/fruit consumption. A plasma sample was analyzed for each participant at each study time point for the following individual carotenoids: α-carotene, β-carotene, lycopene, β-cryptoxanthin, and lutein plus zeaxanthin; these were summed for an estimate of total carotenoids (μmol/L) [26]. In a previous analysis, using change in plasma carotenoid concentration as the gold standard, we identified significant measurement error in two self-reported measures of fruit and vegetable consumption [25]; thus, plasma biomarker was the preferred measure to assess change in fruit and vegetable intake.

#### Physical Activity Measure

The WHEL Study assessed physical activity with nine questions from the Women’s Health Initiative on usual physical activity (recreational walking and light, moderate, and vigorous physical activity) using a standard frequency-by-duration item format [27]. This scale was validated against the Physical Activity Recall (PAR) and accelerometers in the study population [27].

### Statistical Analysis

Chi-square tests were conducted to compare proportions of those with depressive symptoms within age, education, race, BMI (kg/m^2^), physical activity (metabolic equivalent (MET)-hours/week), years between breast cancer diagnosis and study entry, and dietary intake categories. Adherence rates to study assessments (24-h recalls and clinic visits) at years 1 and 4 were calculated by study group and depressive symptom category. Logistic regression models were used to calculate the odds of not completing these assessments by study group and baseline depressive symptom category. As it was expected that baseline dietary pattern would be associated with depressive symptoms in the change analysis, we binarized each dietary component using the following cut-points: 5 servings per day of fruits/vegetables, 25 g/day of fiber, and 27.5 % energy from fat. We conducted tests of associations for both short-term changes (from baseline to year 1) as well as how well that change could be maintained (years 1 to 4). All models were adjusted for age, BMI, and physical activity, and dietary models were stratified by baseline dietary intake. Statistical analysis was performed using SAS 9.3 (SAS Institute Cary, NC, USA).

## Results

### Sample Characteristics

Of the 2817 women included in this study, most were non-Hispanic White (86 %), college graduates (54 %), and over the age of 50 years (64 %). Distribution of BMI categories were 42 % normal weight, 31 % overweight, and 27 % obese. One fifth met the threshold criteria for having depressive symptoms (Table 1). Depressive symptoms (CES-D ≥5) did not differ between study groups and were higher in younger survivors (<50 years of age; *p* = 0.001), women who were obese (BMI ≥30 kg/m^2^; *p* < 0.0001), ethnic minorities (*p* = 0.04), those less physically active (<10 MET-h/week; *p* < 0.0001), and with a recent diagnosis of breast cancer (<2 years since diagnosis compared to 3 or 4 years since diagnosis; *p* < 0.001). Additionally, CES-D scores ≥5 were associated with lower self-reported intake of fruits and vegetables (*p* < 0.0001), lower plasma carotenoid concentration (*p* < 0.01), and a higher percent of energy from fat (*p* = 0.03). Depressive symptoms were not associated with education or fiber intake. We examined trends in depressive symptoms over time (data not shown). The prevalence of depressive symptoms declined in the intervention group in the first year by −3.7 % (*p* = 0.03) but reverted to baseline levels in year 4 (*p* = 0.56). No such change was observed in the comparison group.

**Table 1:** WHEL Study participant characteristics by baseline 6-item CES-D category (%). CES-D scores ≥5 indicate depressive symptoms

	Number	CES-D score		*p* value
		<5	≥5	
Study group				
Intervention	1398	79	21	0.77
Comparison	1419	80	20	
Age (years)				
<50	1010	76	24	0.001*
≥50	1807	81	19	
Education				
Never attended college	424	78	22	0.19
Some college	866	78	22	
College graduate or higher	1527	81	19	
Race				
Non-Hispanic White	2409	80	20	0.04*
Other	408	75	25	
BMI (kg/m^2^)				
<30	2081	82	19	<0.0001*
≥30	736	73	27	
Physical activity^a^ (MET-hours/week)				
<10	1348	75	25	<0.0001*
≥10	1469	83	17	
Breast cancer diagnosis to study entry (years)				
<1	624	76	24	0.003*
1–2	911	77	23	
2–3	701	83	17	
≥3	578	82	18	
Plasma carotenoid concentration (μmol/L)				
<1.99 (below median)	1416	77	23	0.006*
≥1.99 (above median)	1401	81	19	
Study targets				
Fruit and vegetable (servings/day)				
<5	715	74	26	<0.0001*
≥5	2102	81	19	
Fiber (g/day)				
>25	726	81	19	0.29
<25	2091	79	21	
% energy from fat				
<27.5	1246	81	19	0.03*
≥27.5	1571	78	22	

*p < 0.05, chi-square significance level

^a^A cutoff of 10 MET-h/week of physical activity was used to approximate the recommended guidelines of 150 min/week

### Completion of Study Assessments and Clinic Visits

As indicated in Table 2, at year 1, 88 % of the intervention group and 93 % of the comparison group completed self-reported dietary assessments with 85 and 87 % completing the clinic measurement visit. In the comparison group, participants with depressive symptoms (compared to those without) had significantly higher odds of not completing dietary assessments [12 vs. 6 %, odds ratio (OR) = 1.94; 95 % confidence interval (CI) = 1.26, 3.00]. There was no such significant difference in the intervention group (13 vs. 12 %). Similar differences in non-completion rates were observed for the first-year clinic measurement visit: comparison group participants with baseline depressive symptoms had higher non-completion rates (19 vs. 12 %, OR = 1.66; 95 % CI = 1.17, 2.34) (Table 2). No such difference was observed in the intervention group (17 vs. 15 %, ns). Similarly, in year 4, comparison group participants with depressive symptoms had significantly higher odds of both non-completion of dietary assessments (18 vs. 9 %, OR = 2.03; 95 % CI = 1.40, 2.97) and clinic visits (22 vs. 15 %, OR = 1.48; CI = 1.06, 2.07). Again, in the intervention group, there was no significant difference by depressive symptoms for non-completion of either dietary assessments (19 vs. 16 %) or clinic visits (25 vs. 22 %).

**Table 2:** Odds of not completing WHEL Study dietary assessments and clinic visits at year 1 and 4 follow-up by baseline six-item CES-D cutoff scores of ≥5, adjusted for age, BMI, and physical activity

	Number	Dietary assessments		Clinic visits	
		% not completed	OR (95 % CI)	% not completed	OR (95 % CI)
Year 1					
Intervention					
CES-D <5	1106	12		15	
CES-D ≥5	292	13	1.06 (0.72–1.57)	17	1.00 (0.70–1.43)
Comparison					
CES-D <5	1129	6		12	
CES-D ≥5	290	12	1.94 (1.26–3.00)*	19	1.66 (1.17–2.34)*
Year 4					
Intervention					
CES-D <5	1012	16		22	
CES-D ≥5	275	19	1.02 (0.72–1.46)	25	1.01 (0.73–1.40)
Comparison					
CES-D <5	1028	9		15	
CES-D ≥5	274	18	2.03 (1.40–2.97)*	22	1.48 (1.06–2.07)*

*CES-D* Center for Epidemiologic Studies Depression Scale (≥5 indicates depressive symptoms)

*p < 0.05, logistic regression significance level

### Dietary Changes Between Treatment Groups at Years 1 and 4

At baseline, there were no between-group differences in any of the three dietary-related variables (plasma carotenoids, fiber, or energy from fat) (Table 3). For plasma carotenoids, in the intervention group, this biomarker increased by 47.4 % by year 4 compared to an increase of only 6.2 % in the comparison group (*p* < 0.0001). For fiber intake, intake increased by 25.9 % at year 4 in the intervention group while intake decreased by 4.9 % in the comparison group (*p* < 0.0001). For percent energy from fat, at year 4, the intervention group’s estimate decreased slightly whereas that of the comparison group increased by 14.4 % (*p* < 0.0001).

**Table 3:** Mean (SE) total plasma carotenoid concentration, dietary fiber, and percent energy from fat in the WHEL Study intervention and comparison groups

	Mean (SE)		*p* value
	Intervention	Comparison	
Plasma carotenoid concentration (μmol/L)			
Baseline	2.3 (0.04)	2.3 (0.04)	0.33
Year 4	3.2 (0.07)	2.2 (0.04)	<0.0001*
% difference from baseline to year 4	47.7 (3.0)	6.2 (1.5)	<0.0001*
Fiber (g/day)			
Baseline	21.2 (0.2)	21.2 (0.2)	0.98
Year 4	25.2 (0.3)	19.3 (0.2)	<0.0001*
% difference from baseline to year 4	25.9 (1.6)	−4.9 (1.0)	<0.0001*
Percent energy from fat			
Baseline	28.4 (0.2)	28.7 (0.2)	0.34
Year 4	27.2 (0.2)	31.4 (0.2)	<0.0001*
% difference from baseline to year 4	−0.0 (1.0)	14.4 (1.0)	<0.0001*

*p < 0.05, two-sample *t* test significance level

### Change in Dietary Targets by Depressive Symptoms at Years 1 and 4

We present 1- and 4-year changes in these three dietary variables by two levels of baseline intake (closer to vs. further from the study dietary target) and by whether participants had baseline depressive symptoms or not (Table 4). Among those with better vegetable/fruit intake at baseline, there was no difference in the increase in plasma carotenoid concentration by baseline depressive symptoms (75.5 vs. 73.2 %, *p* = 0.54), nor was there an association in the percent decline in carotenoids between years 1 and 4. Similarly, for those with higher baseline fiber consumption, baseline depressive symptoms were not associated with either 1- or 4-year change. Among those who consumed less fat at baseline, depressive symptoms were not associated with decreases achieved in the first year; however, it was associated with the increase observed between years 1 and 4 (+37.9 vs. +27.3 %, *p* = 0.04).

**Table 4:** Mean (SE) percent change of dietary targets from baseline to year 1 and from years 1 to 4 among WHEL Study intervention participants without (<5 CES-D) and with (≥5 CES-D) depressive symptoms, by baseline dietary intake

	Number	<5 CES-D	Number	≥5 CES-D	*p* value
Baseline to year 1					
Plasma carotenoids (μmol/L)^a^					
<5 fruit-vegetable servings/d	210	69.7 (6.9)	72	37.2 (7.2)	0.02*
≥5 fruit-vegetable servings/d	717	75.5 (3.3)	166	73.2 (8.2)	0.54
Fiber (g/day)					
<25	723	61.2 (2.2)	186	49.0 (4.2)	<0.01*
≥25	253	12.8 (1.9)	67	9.2 (4.6)	0.57
% energy from fat (per day)					
<27.5	449	−6.6 (1.4)	105	−8.0 (3.3)	0.73
≥27.5	527	−26.7 (0.9)	148	−22.4 (1.8)	0.02*
Years 1 to 4					
Plasma carotenoids (μmol/L)					
<5 fruit-vegetable servings/d	153	−6.1 (3.8)	56	−7.3 (5.1)	0.34
≥5 fruit-vegetable servings/d	557	−7.9 (1.7)	130	−6.6 (4.6)	0.76
Fiber (g/day)					
<25	601	−11.4 (1.2)	161	−11.6 (2.6)	0.86
≥25	218	−7.8 (1.7)	58	−9.5 (3.5)	0.87
% energy from fat (per day)					
<27.5	376	27.3 (2.0)	88	37.9 (5.9)	0.04*
≥27.5	443	24.7 (1.9)	131	23.7 (3.7)	0.68

*p < 0.05, ANCOVA significance level adjusted for baseline dietary intake, age, BMI, and physical activity

^a^Percent changes in plasma carotenoid concentration (a biomarker of fruit and vegetable consumption) categorized by fruit and vegetable intake according to baseline 24-h recalls

A different pattern was observed in those who were further from the study’s targeted dietary pattern at baseline. Baseline depressive symptoms did make a difference in the amount of change achieved in the first year, but there was no difference in the degree of relapse seen between years 1 and 4. For those with low baseline vegetable intake, the first-year increase in plasma carotenoid concentration was lower for those with baseline depressive symptoms than that for those without (37.2 vs. 69.7 %, *p* = 0.02). Similarly, in the first year, baseline depressive symptoms were associated with smaller increases in fiber consumption (+49 vs. +61.2 %, *p* < 0.01) and smaller decreases in energy consumed from fat (−22.4 vs. −26.7, *p* = 0.02).

## Discussion

The WHEL Study randomized participants to an intensive intervention aimed at achieving a major long-term change in diet that could reduce their risk of a number of health conditions, including their risk of recurrence from breast cancer (which the study was testing). We confirmed that those with baseline depressive symptoms had poorer diet than those without such symptoms [5]. Further, we confirmed that depressive symptoms at baseline were associated with lower levels of engagement with key study measurements, at least in the comparison group [6]. However, the study’s external incentive program appears to have raised the engagement level of all participants so that the observed differences were unlikely to confound study results.

The study intervention, with its behavioral activation components, achieved a major change in each of the dietary variables during the first year in those with and without depressive symptoms at baseline. A difference in the level of change achieved in the first year by depressive symptom status was only observed in the subgroup that was further from study targets. Of interest was the lack of a depressive symptom effect on relapse between years 1 and 4, except for energy from fat among those with the better dietary pattern at baseline. We have previously identified that fat intake in the study increased over time, apparently following the national diffusion of the popular Atkins diet [28]. Participants with low intakes of energy from fat at baseline increased their fat intake more if they had baseline depressive symptoms. Such an effect might be expected if the depressive symptoms were related to body weight. However, overall, while we were able to identify the expected differences by depressive symptoms in this study, the differences did not appear to be large enough to seriously confound the study conclusions. The inverse relationship between depressive symptoms and engagement level in the comparison group was consistent with other studies [2, 20, 29].

One possible explanation for the observed effect of depressive symptoms is that participants with these symptoms were more likely to have had *ruminative responses*, which involve repetitively and passively thinking about negative emotions that interfere with other activities despite the prospects of extrinsic rewards [30]. Behavioral activation aims to disrupt this cognitive pattern [31, 32] by setting out to increase positive reinforcement [33, 34]. The WHEL dietary intervention consisted of several behavioral activation components. The initial presentation aimed to build participants’ motivation to make dietary changes that were hypothesized to reduce cancer recurrence. Additionally, the telephone counseling component was highly client centered: counselors worked closely with participants to set challenging but achievable goals and provided frequent positive feedback on their performance. Thus, the intervention provided many opportunities to increase the number of reinforcing events. This intervention mitigated the inverse relationship between depressive symptoms and critical study assessments (completing dietary assessments and attending clinic visits) in both the short (year 1) and longer (year 4) term. If adding behavioral activation to the intervention was sufficient to remove the depressive symptoms’ effect on adherence, and then we would also expect that the intervention group would have reduced prevalence of depressive symptoms over time. Such an effect was observed, at least during the first-year intensive component of the intervention.

Another finding in this study deserves comment. While the intervention removed the discrepancy on adherence from depressive symptoms, it did not achieve the high levels of adherence seen in the comparison group without depressive symptoms. It is possible that the intensity of the WHEL intervention may have created additional participant burden that resulted in reduced participation in assessments. Alternatively, as the comparison group had much less contact with team members during the study, they may have been more motivated by the study’s extrinsic rewards aimed at maximizing adherence to study measures. Further research is needed to explore the effect of intensive interventions on adherence to study measurement.

The equivalent completion rates for assessment between those with and without depressive symptoms in the intervention group created an ideal setting to compare the relative effectiveness of the intervention among those with and without depressive symptoms at baseline. First, the intervention group achieved and maintained some of the largest levels of change reported particularly in vegetables, fruit, and fiber [35]. For those who needed to make the least change to achieve study targets, there was no association of depressive symptoms with the level of dietary change achieved. However, for the subpopulation whose baseline diet was furthest from study targets, the amount of change achieved differed by depressive symptom level, although even those with depressive symptoms made major changes in their dietary pattern. Irrespective of the baseline depressive symptom status, the study intervention was able to maintain the changes achieved by year 1 through year 4.

The WHEL Study sample consisted of women who were mostly over the age of 50 and, therefore, in one of the population groups most likely to have depressive symptoms. The generalizability of study results is limited as all participants had been diagnosed with invasive breast cancer, and they had volunteered for a nutrition study that had the potential to reduce their risk for a breast cancer recurrence. However, in this study, depressive symptoms were associated with less healthy lifestyles and not with breast cancer characteristics [15], which suggests that the findings have relevance to a wider population. The study used a six-item self-reported depressive symptom instrument that has been shown to have both good sensitivity and specificity, although the positive predictive value is less impressive [24]. However, some of the symptoms measured also reflect anxiety and psychological distress [21], so the label of depressive symptoms may be too narrow to reflect what was actually measured.

A noted strength is that this study is a randomized controlled trial with a large number of participants and a significant subsample with depressive symptoms. Another major strength was the availability of a biological measure of dietary intake, plasma carotenoid concentrations, to assess fruit and vegetable intake, which was a major behavior change targeted in the WHEL intervention. The study used multiple measures of adherence (i.e., completion of assessments, clinic attendance, and dietary behavior), for which there was generally a stable inverse relationship with depressive symptoms. However, the very high completion of assessments and clinic attendance were likely a consequence of using an innovative system of extrinsic rewards/incentives. These findings support that assessment of baseline depressive symptoms in dietary intervention studies may reduce potential confounding and enhance generalizability [2]. They also support the inclusion of behavioral activation and other intervention strategies aimed at minimizing the effect that depressive symptoms could have on study results.

## Conclusions

This study found that baseline depressive symptoms were associated with lower adherence to study assessments in the comparison group, although the study’s extrinsic reward system ensured that all groups had high adherence. The study intervention included a number of components that can be characterized as behavioral activation strategies, and these were associated with an apparent removal of the expected depressive symptoms effect that was limited to the intensive intervention phase. The intervention was highly successful in encouraging participants to make major changes to their dietary pattern, particularly among women farthest from the dietary targets at baseline. However, the intervention effect on dietary changes was less pronounced among women with depressive symptoms. These findings suggest that future studies can identify their subpopulation with depressive symptoms and ensure that their intervention includes strategies such as behavioral activation that can reduce the possible impact of these symptoms on study measurement and outcomes.
